# “I Am Very, Very Proud of Myself”: Improving Youth Activity Levels Using Self-Determination Theory in Program Development

**DOI:** 10.3389/fpubh.2013.00046

**Published:** 2013-10-29

**Authors:** Judy B. Springer

**Affiliations:** ^1^Physical Education Department, Milwaukee Area Technical College, Milwaukee, WI, USA

**Keywords:** physical activity youth, theory-based behavioral interventions, community-based, self-determination theory, psychological need satisfaction

## Abstract

Many adolescents are not meeting recommended levels for physical activity. Increasing physical activity among urban African American youth is both a challenge and a public health priority. Most research in community-based interventions has taken a didactic approach, focusing on skill and knowledge development alone, with inconclusive results. This 10-week progressive activity intervention with adolescents in an urban faith community introduced a self-determination theory (SDT) approach with the aim of promoting the adoption of self-management skills necessary for sustaining activity. Components of SDT included relatedness, competence, and autonomy. Together with didactics, aligning activities with participant interests, and using existing social structures for health message delivery, the approach led to high satisfaction ratings for the three components of SDT along with improved skills, knowledge, and outcomes in cardiovascular fitness. Understanding and utilizing approaches that enhance enjoyment, personal choice, confidence, and social affiliation may lead to more lasting healthy activity behaviors and attitudes than didactic approaches alone in this and other adolescent populations. The SDT is reviewed in the context of this youth intervention.

## Introduction

Physical inactivity is a major public health concern in the United States that affects adolescents as well as adults. By ninth grade nearly 80% of teenagers do not meet physical activity recommendations, and their activity levels decline steadily with age, particularly among girls (1). Differences in physical activity appear as a function of race, socioeconomic status, and gender (2). These differences place children and adolescents from minority and low socioeconomic groups at risk for disparities that will affect their lifelong health and well-being; increasing physical activity is therefore a vital health priority. In 2011, 81% of 9–12th grade students in Milwaukee, Wisconsin, did not participate in recommended levels of physical activity. African American females had the lowest levels, with 89% failing to meet physical activity guidelines (1). The predominantly African American Amani neighborhood in which the present study was conducted has the lowest socioeconomic status in the city, with nearly half the households in poverty and headed by jobless adults (3), making it a desirable place to locate an activity intervention.

Public health scholars and practitioners want to identify and understand strategies that will promote youth physical activity. No studies to date have examined the components of relatedness, competence and autonomy in the design of youth activity programs. Two studies looking at community-based interventions for increasing activity levels in youth included only an educational component, and results were inconclusive (4, 5). The main aim of this study was to determine the influence of programing framed in self-determination theory (SDT) constructs for adolescents’ physical activity engagement in an urban faith community.

### Self-determination theory

Self-determination theory is an organismic-dialectic framework of motivation that considers humans to be actively seeking optimal challenges and new experiences to master and integrate in their internal and external environments (6–9). SDT suggests that the satisfaction of basic psychological needs promotes healthy development (10).

Deci and Ryan (9) hypothesized three psychological needs fostering this self-motivated process: relatedness, competence, and autonomy. Relatedness is feeling connected to and valued by significant others in a given social context; competence is feeling that one can achieve behaviors and reach goals; and autonomy is feeling that behavior originates in the self (11). According to Deci and Ryan (12), although people have an inherent tendency toward maintaining their well-being, this natural predisposition diminishes when these three needs are not met (13). However, certain conditions and strategies foster fulfillment of these needs. Relatedness is fostered when individuals are provided with a sense of connection and belonging with others who engage in the activity with them. Competence is developed through experiences that challenge individuals to use their skills but is not supported when extremely easy or difficult challenges are presented to the individual (13). Autonomy arises when people are offered choices and provided relevant information with minimal pressure and control. In environments that provide for these needs, individuals are more likely to be self-motivated to sustain the desired activities (14). The individual continues in the activity because of both intrinsic satisfaction (i.e., enjoyment, challenge, connection) and well-internalized extrinsic motivation (e.g., improved appearance or health), leading to an internalized value in the activity (13).

An examination of the use of SDT in programs with adults in community settings is worthwhile as this information may be related to programs with youth. When individuals focused on enjoyment, competence, and social interaction for physical activity, adherence rose at a weight-training center (26), whereas the need for competence was the main predictor of physical activity attendance in organized group fitness programs (27). However, in two separate studies, fulfillment of all three basic psychological needs (competence, relatedness, and autonomy) was associated with participation in a 10-week physical activity class (28) and in a structured 7-week worksite physical activity program (29).

To date, little research has addressed the basic need satisfaction aspect of SDT in the context of youth physical activity or applied the principles of SDT in such programs. Strategies may include: offering a clear rationale for the adoption of the behavior and building sustainable knowledge that supports informed choices may be an SDT principle relevant to youth programs (16); providing positive feedback as a verbal reward, which usually enhances intrinsic motivation because it affirms personal competence (30); offering a menu of options for leisure-time physical activity outside the program may lead to greater sustainability of activity (31); promoting opportunities for socialization, shared problem solving and teamwork have a strong positive influence on psychological need satisfaction (32); practicing skills necessary for completion of specific tasks, such as exercising at a given intensity or reading food labels, promotes competence (33) and, finally, providing well-defined incentives for participation in the initial stages may lead to sustained involvement (34) (Table 1). Applying these principles aim to promote the adoption of self-management skills for sustained activity.

**Table 1:** **Program activities to promote relatedness, competence, and autonomy**.

Activity	Description	Relatedness	Competence	Autonomy
**CHECK-IN/ACCUMULATED MINUTES**				
Accumulated minutes outside of session with incentives	Youth are provided information on what constitutes physical activity, choose activities of interest and skill level, and are awarded incentives for accumulated minutes of activity (15, 16)		XX	XX
**WARM-UP: STRETCHING AND TEAM BUILDING**				
Group stretch by gender	Flexibility is enhanced through static and dynamic stretching lead by program staff; groups are designated by gender (17)	XX		
Connection	Game allows participants to see similarities and differences with others with the ultimate goal to “connect” into a large circle (18)	XX		
Hula hoop pass	Participants form a circle, join hands with two individuals letting go just long enough to place hands through a hula hoop; everyone works together to pass the hoop around the circle without disconnecting hands to promote teamwork and communication (19)	XX	XX	
Double Dutch jump rope	A style of jumping rope where there are two participants turning two ropes while one or two participants jump through the ropes (20)			
**RUN/WALK SEGMENT**				
Progressive run/walk segments	Activity is self-paced in a group setting; goal is to keep moving; intensity is based on perception of effort by participant (21, 22)		XX	XX
Partner run with discovery questions	Pairs of youth run/walk together to adjust to steady and natural pace; youth get to know partner (23)	XX	XX	
Ladder run/walk	Team of four to five youth form single-file line, maintain steady pace; last person in line sprints to front to become leader and set pace; team maintains pace to prevent last person from falling “off the ladder”		XX	XX
**EDUCATIONAL SEGMENT**				
“What runners need” interactive game	Focusing on nutrition for performance participants review foods that are healthy choices for athletes and those that reduce performance; information for parents provided (23)		XX	XX
Running form	Allowing for individual differences proper running technique is provided and reinforced in subsequent sessions with youth (24)		XX	XX
Nutrition label reading	Understanding nutrients in foods can help youth use the Nutrition Facts Label more effectively, and enable them to make choices that best suit their own health needs; many real world examples provided (25)		XX	XX

## Materials and Methods

The study was reviewed and approved by the Institutional Review Board at Milwaukee Area Technical College and the Medical College of Wisconsin.

### People and settings

The research team consisted of two senior health researchers (Ph.D.), an advanced nurse practitioner and several trained research assistants. Health care professionals recruited from the community and local community college service-learning students were also involved in the intervention. A convenience sample of parents of middle- and high-school youth in an urban church in Milwaukee, Wisconsin, received program brochures written by program staff and sent by the church youth minister and youth workers. Interested families contacted the youth minister to initiate recruitment process. Pre-intervention Health History Screening Questionnaires (35) were administered by program staff to determine any limitations for physical activity. Program activities were held during regularly scheduled Wednesday evening Bible study at a YMCA site centrally located to the church. A 5 km community road race was held locally.

### Instruments

#### Training effect

Cardiorespiratory intensity was self-rated and reported during physical activity using the OMNI Rating of Perceived Exertion (RPE). The OMNI scale is a psychophysical estimation method to self-report overall feelings of exertion, or in the case of youth, “tiredness,” which correlates strongly with increased heart rate and respiration during physical activity. The RPE scale is used in predicting fitness, clinical and field settings, and exercise prescription (22). Changes in RPE over the course of a training program indicate an improvement in fitness level. Analysis consisted of paired *t*-tests of collected data.

#### Surveys, observations, debriefing, interviews

Surveys were completed by youth to rate gains in skills and knowledge, and evaluate satisfaction with program variables. We also performed post-program self-rated assessments, observation/debriefing, and individual elicitation interviews. The survey instruments were developed by program staff. One contained seven-questions the seven-questions, both Likert-type and open-ended questions to gauge youth program satisfaction, interests, and potential program adaptations. A six question post-training self-rated assessment aimed at program objectives: learning and healthy lifestyle practice acquisition.

Debriefing sessions were held after each session with program staff and with trained lay leaders in weeks 1, 5 and post-program to consider program successes and needs for modification. The senior researcher kept field notes of each session content.

A senior researcher with experience in qualitative assessment techniques conducted the open-ended interviews with youth to enable participants to describe their experiences with specific aspects of the intervention.

### Intervention

The 10-week youth running sessions with incentives for participation have been detailed elsewhere (36). Briefly, the 90-min session included: 5 min for organizational tasks; 10 min for warm-up including walking and stretching; 20 min for game and team building; a 30-min opportunity for run/walk session during which participants were encouraged to remain constantly active; 5 min for cool-down including light stretching; and 20 min for the didactic session. Running-related games such as a ladder run or partner run were used to promote full participation. Program staff participated alongside youth and explained and recorded youth OMNI RPE Scale (22) during the run segments of weeks 1, 5, 7, and 10. Participants also provided pre- to post-program self-rated assessments and engaged in open-ended and session surveys.

Educational sessions followed physical activity and included healthy snacks and hydration. Topics focused on improving knowledge of physical activity guidelines, healthy lifestyle practices, and exploring the link between spirituality and health (Table 2). Each didactic session was facilitated by program staff, health care professionals recruited from the community and local community college service-learning students with interactive games and worksheets provided to reinforce main concepts. Program staff developed a one-page synopsis of each session that was sent home to parents to maintain their interest in the program and aid them in establishing healthy habits for their family.

**Table 2:** **Select youth educational topics**.

Session	Topic(s)
1	Importance of physical activity for health
2	Choosing foods for fuel as a runner
3	Building a healthy, spiritual foundation/recipe for a spiritual life
5	Components of proper running form
7	Nutrition label reading and USDA dietary guidelines
8	Tips for success in high school as a scholar and athlete
10	Future steps for health

To promote youth leisure-time physical activity outside the program in accordance with national physical activity recommendations (37), two methods were used: following a didactic session explaining the guidelines a series of incentives for self-reported accumulated minutes of physical activity was created, and participation in a local 5 km run/walk event was promoted. Using specially designed “Physical Activity Trackers,” youth identified and recorded moderate- to vigorous-intensity physical activities with incentives awarded bi-weekly based on accumulated minutes. To promote engagement with the local community in physical activity, 14 youth and 6 program staff registered for a local 5-km (3.1 miles) run/walk event.

### Analyses

In this study, we used session surveys, post-program self-rated assessments, observations, and interviews to develop an understanding of youths’ perceptions of factors associated with involvement in a structured 10-week youth run/walk program. Through the use of multiple sources of data collection, we were able to triangulate the data in such a way that improved the trustworthiness of information (38, 39).

#### Qualitative analysis

All survey and assessment results, observations, interviews, and field notes were transcribed. Analysis focused on self-determination-based constructs and used basic principles of qualitative data analysis (40). Starting with the survey data, we began developing an overall coding scheme and then used the observations and interviews to both expand upon and refine the codes. Employing an iterative approach, a team member analyzed the data for themes with open-coding and began to develop an overall coding scheme by using additional data to refine the codes. The senior researcher made the final decision on codes, categories, and themes.

## Results

Of the 35 youth recruited, complete data were available for 24 middle- and high-school youth [14 female, 10 male (mean age 13.5 ± 1.98 years, Body Mass Index (BMI) 26.5 ± 5.94)]. Eleven did not enter the program, eight because of challenges associated with paperwork completion and three because of lack of interest after being informed about the study.

Based on responses to the Health History Screening Questionnaire, no youth had medical conditions requiring physician permission for physical activity. Pre-existing conditions that may impact physical activity participation included asthma and/or allergies. For the 14 completers (5 female, 9 male age was 14 ± 1.96 years, BMI 25.7 ± 6.68) who attended at least 70% of all sessions, average attendance was 8 of 10 sessions. Four of the 14 completers participated in some type of sport activity (track and rugby = 1, football = 2, track = 1) while 1 of the 10 at-large youth participated in volleyball.

### Cardiovascular fitness

At regularly scheduled intervals of the run, youth were asked to provide self-rated RPE values. The collected OMNI RPE values indicated two important findings. First, over time the 14 completers (those attending at least 7 of the 10 sessions) experienced a training effect with each conditioning session (progressive run/walk interval) with RPE-approaching-5 rating, which reflects an understood ventilatory breakpoint (22). Second, the RPE values from pre- to post-test suggested improved cardiorespiratory endurance across time for the youth. From Week 5 (run/walk = 2/1 min) to Week 10 (run/walk = 4/1 min), RPE value differences were statistically significant (paired *t*-test, *p* < 0.05).

### Self-rated assessment

**Figure 1:**
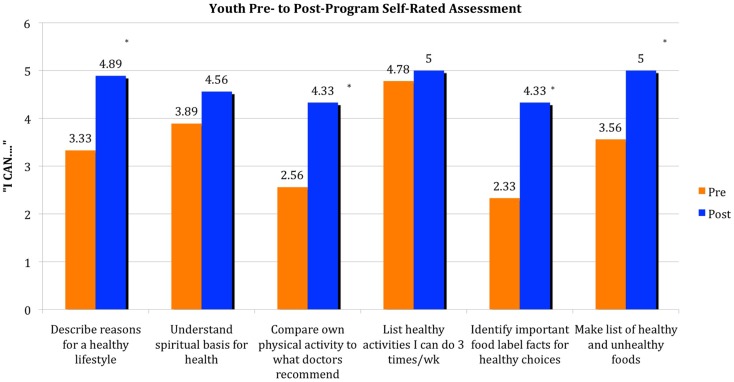
**Youth (*n* = 9) were asked to preview (week 1) and complete (week 10) a pre- to post-program self-rated assessment of skills associated with physical activity and nutrition**. Statements began with “I can … ” and corresponded with the program’s objectives. Results are based on a Likert-type scale 1–6, 1 being “not at all – I don’t know anything about this and cannot do this” to 6 being “very much – I know a lot about this and have experience doing this.” Key: *denotes *p* < 0.01.

Pre- to post-program self-rated assessments collected in weeks 1 and 10 are included in the Figure 1. Results seem to indicate an increase in awareness of healthy lifestyle practices for these youth, especially knowledge related to an increased awareness of reasons for a healthy lifestyle that includes regular physical activity and the ability to compare current individual physical activity levels to what doctors recommend for individuals of their age. Gains in association with the program objectives related to nutrition occurred for youth who reported an increase in knowledge and skills for identifying important food label facts for healthy choices and ability to make a list of healthy and unhealthy food choices (*p* < 0.01). Youth showed small improvements in other program objectives (e.g., understanding the spiritual basis for health) or may have previously had a strong knowledge base that did not necessarily translate in to action (e.g., list types of physical activity that can be performed three times per week).

### Program engagement

Based on the multiple sources of data, we provide information and examples of the evidence we used to determine whether the participants were physically and psychologically engaged in the physical activity intervention. Accordingly we elicited responses and behaviors indicative of SDT constructs. Statements descriptive of theory categories are included: (1) Relatedness – *I like it here* (belonging, teamwork); (2) competence – *I liked that I ran and actually kept up* (skill acquisition, accomplishment, understanding health); and (3) autonomy – *The best thing I did was the running* (strategies for lifelong health, testing new skills).

### Relatedness (*I like it here*: Belonging, teamwork)

Having a place to be with peers while performing an activity that was neither threatening nor competitive appears to have provided a sense of belonging. For example, in high demand were games such as Double Dutch jump rope, especially with the involvement of lay leaders in teaching unique techniques, and team activities where learning a new skill led to learning new ways to relate to each other. “Thanks for allowing me to be a part of the program. I am enjoying myself with all the games,” said a male, age 14 years. Youth reflected on the importance of the environment for maintaining the program effectively and had strong buy-in through their participation and enjoyment in the initiative (Table 3). While program staff encouraged youth to go at their own pace, we observed youth cooperatively encouraging each other to push a little harder to complete the run segment. They also evaluated their own performance based on their own perception of effort. By programs’ end we observed youth who had initially tended to keep to themselves (e.g., walking the perimeter of the gym alone) become more receptive to other youth and more fully engaged in all activities (e.g., participating in problem solving within teams). Youth actively engaged with program staff by sharing stories and anecdotes from throughout their day. Teamwork and problem solving were common elements in games such as the hula hoop pass where youth worked as a team to pass the hoop, requiring cooperation to complete the task successfully. Acquiring team shirts for all participants was especially important in developing relatedness in the 5 km community run/walk event. “There were so many people at the run. I knew I was okay as long as I saw other team members with a purple shirt” (female, age 11).

**Table 3:** **Youth responses to survey instrument**.

Session	One	Three	Four	Five	Six	Seven	Eight	Nine	Ten
Did you like today’s topics and people who presented information?	3.80	3.88	3.63	3.21	3.47	3.54	3.59	3.45	3.89
Did you like the activities/walking and running?	3.50	3.53	3.00	3.21	3.40	3.00	3.47	3.17	3.57
Do you feel confident, that in the next week, you’ll use healthy ideas presented by the program?	3.80	3.53	3.53	3.16	3.33	3.31	3.65	3.56	3.63
Overall, did you like today’s session?	3.70	3.59	3.42	3.26	3.40	3.51	3.53	3.44	3.64
Number of respondents	10	17	19	19	15	13	17	18	14

*Key: Likert-type scale, 4 = yes, very much; 3 = it/they were good; 2 = it/they were okay; 1 = no, not at all. Session Two data not available*.

Relatedness also played a role in the competitive nature of team play as youth were challenged in relay races and on the 5 km run/walk. When asked for a general sense of how he was doing, a male, age 15 responded, “I’m doing fine as long as I am in the lead of the purple shirts.” Creative strategies that leaders used to engage youth in the application of teamwork principles included assigning youth to team-up with others who appeared less fit or shy to demonstrate leadership, empathy and cooperation, providing opportunities for shared problem solving in skill-building games and encouraging friendly competition (e.g., males versus females, older versus younger participants).

### Competence (*I liked that I ran and actually kept up*): Skill acquisition, accomplishment, understanding health

#### Skill acquisition

Youth and program staff reported many examples of program components that enhanced the ability to perform physical activity, including instruction in maintaining proper running form. While running and walking may be viewed as simple activities that do not require a special skill set such as those needed in soccer or basketball, the Running Form session was viewed by youth as important for skill development and confidence in completing the task and led to the majority (81%) of youth stating the segment was the best part of the evening: “In this session I learned how to run *properly*, which will make it easier for me to run longer,” a male, age 17, noted. Other youth commented that learning how to run better made the activity more manageable and increased the likelihood that they would reach the suggested goal. “The best thing I did was push myself to run the amount of minutes given,” said a female, age 16. We observed youth focusing on proper technique during each session and at the community run/walk event.

#### Sense of accomplishment

Initial youth comments centered around strategies for building endurance including preventing cramping and being able to run for longer duration. With each session the overall sense of accomplishment emerged for these youth. For example, being able to complete the specified number of minutes without stopping resulting in feelings of satisfaction and accomplishment. A female, age 13, shared, “I’m getting better because I’m running so fast now. I am very, very proud of myself.”

A single case provides a glimpse into the sense of accomplishment conveyed by the youth. John (pseudonym), male, age 13, a basketball player, attended 8 of 10 sessions, received numerous incentives, and successfully completed the community 5 km run/walk event. John noted:

After a lifetime of failure I have finally accomplished something, and it was exercise. If anyone would have told me that I would be able to run three miles I would have thought they were crazy. Now that I did it, it was really okay. When I went to school on Monday my friends were giving me a hard time about how I was walking all stiff and funny. I told them that I had done a 3-mile race over the weekend. They shut right up. They couldn’t believe, and I couldn’t either, that I had done the whole thing. It was really great!

#### Understanding health

During interviews and through survey information, participants attributed improved health awareness and health knowledge to participation in the youth program. For example, several participants reported that they were more likely to engage in physical activity for longer periods of time to improve their health: “I know now that if I am active for 30 min it will raise up my health.” While the structured program provided an outlet for activity during the session, youth inquired what other activities might be considered exercise (e.g., playing games with little brothers and sisters, doing the laundry). Prior to the program many youth considered only activity done in a gymnasium to count toward health. They were encouraged to learn that many life activities constitute physical activity. A 17-year-old male “was surprised to learn that many of the things I do every day are considered exercise. I know now that I can regularly do activities to be more healthy.”

Others spoke of finding ways to be active at home or school by taking the stairs or walking in the hallways for improved health. Based on self-rated assessments youth reported gains in knowledge on what constitutes a healthy lifestyle and on what a doctor would say is the right amount of physical activity for them (see Figure 1).

Nutrition was another area of health in which youth gained competence in being able to choose healthier snacks and obtain important nutrient information. Responses to the educational component (i.e., the presentations on various health topics and the accompanying handouts) were largely positive. Information on making healthy choices by nutrition label reading was very well received. A male, age 17, wrote, “Tonight I learned that taco salad is 820 calories so stay away.” Others reflected on a new skill of being able to read calories on a nutrition label as a great way to choose healthier foods and “watch what I eat.” Each week a nutritious snack was provided to the youth to reinforce in their actions what was emphasized in multiple messages: good nutrition is important for optimal performance as a runner and walker and can be achieved through simple but intentional strategies.

### Autonomy (*The best thing I did was the running*): Strategies for lifelong health, testing new skills

Youth reported a sense of satisfaction in being able to choose the duration and set their own pace during the run/walk sessions and to lead peers as positive role models in healthy behaviors. During the run segments we observed youth being encouraged to keep moving, meaning the individual could work at his/her own pace as long as he/she kept moving. While some youth appeared to naturally work into a rhythm of activity with peers, the opportunity to be active at will without penalty was helpful. Youth appeared to motivate from within when given opportunity to set their own pace. “The best thing I did was pushing myself to run the amount of minutes given,” said a female, age 16. As youth become more comfortable with the run/walk routine they were able to be an example to others for healthy behaviors. Many participants suggested that pushing themselves to go just a bit further by partnering with others or maintain the pace for a while longer was important for success. One 13-year-old female commented about the 5 km community run: “… I wasn’t really sure who to run with. I saw a girl who was about my size and about my age. I was able to run with her, in fact we ran just about the whole race together. It felt great to work together to accomplish our goal. We also saw others from our program who encouraged us to keep going.”

The program provided a venue for trying new activities, learning new skills, and was supportive of exploration in physical activity for these youth. A feature of the program to promote leisure-time physical activity was providing the youth with a physical activity tracker to record accumulated minutes of activity for incentive prizes. At first youth struggled with how to record the activity and what types of activities could be used as exercise. Program adaptations were made to provide additional instruction via community college service-learning students and program staff. As a few youth began recording minutes and receiving incentives, more youth became involved suggesting the process became much more enticing and manageable. While the information from the reported minutes was inconsistent, for those recording minutes, average weekly minutes were reported in most cases. However, only a few youth reached recommended levels of leisure-time physical activity.

A less often discussed component of health was covered in this faith-community-housed program: the responsibility to maintain good physical health as an extension of spiritual life. Youth reported high satisfaction in the exploration of principles of health and their relation to spirituality. The session where a spiritual recipe for health was discussed was the most highly rated (3.88 on of a scale of 4) of all sessions by the youth. The youth minister explored the subject of good health as a necessary action for genuine spirituality by building a life “cake” with ingredients (e.g., flour, salt, whole cream, sour milk) to either improve or deter from the taste of the cake. We observed youth engaged in a meaningful way when the message of spirituality and health was presented in a concrete way with an actual ingredient demonstration to reinforce the theme. The result of the session was the observation by a 15-year-old male, “Spirituality is key to living a healthy life.” Youth also reported high confidence in being able to apply at least one healthy idea from the spirituality and health lesson. A female, age 16, stated, “The cake experiment helped me to better understand my spiritual ways. I love this class; it helps me know how to do better.” The power of the initiative in the faith community was apparent in the manner the youth recognized their role in making good choices for maintaining their health.

The skill acquisition component was also evident in youth learning and participating fully in games of skill and problem solving. An example includes the high interest, enthusiasm, and participation in the games where physical and cognitive prowess was combined. Through numerous observations the interest of trained lay leaders was instrumental in encouraging the youth to test out new techniques in learning the games with their peers. Youth reported that they really liked coming to the program where the leaders showed interest in their advancement, the opportunity to interact with others from their peer group, and be with supportive others.

## Discussion

To our knowledge this is the first study to use a SDT-based intervention with middle- and high-school youth to look at the influence of program factors such as relatedness, competence, and autonomy on participant engagement in physical activity and healthy lifestyle practices. While success in the overall program was defined as improved cardiovascular endurance and attainment of knowledge, the qualitative perspective of this study looked to factors affecting the change in behavior, knowledge, and personal commitment necessary to start and remain engaged in healthy lifestyle choices.

Notably, the findings support findings that the presence of peers and friends is associated with greater enjoyment of physical activity and is a powerful motivator for continued participation. Although many investigators have examined peers as a moderator of social and emotional development (41), our findings suggest that physical activity in a group setting provides a venue for social support, thereby increasing participation. Friendships offer important opportunities for companionship and physically active alternatives to eating and sedentary behavior (42). Whereas the majority of attention has been devoted to the study of the ways in which peer relationships impact cognitive, psychological, and emotional development (41), peer relationships are also relevant for understanding youths’ involvement in active leisure and recreational activities. With their peers, youths engage in team sports and other physical and leisure activities that provide the context for the development of physical abilities and socio-emotional competencies. Finding ways for youth to engage in pleasurable, health-building activities with friends may lead to sustainability of improved health outcomes.

In order for youth to improve cardiovascular fitness leading to improved health outcomes, they must perform sustained activity. The current findings suggest an increased level of endurance was due in part to increased competence, or exercise self-efficacy, in these youth. Because perceived exertion (43) and self-efficacy, meaning the sense of confidence in personal physical activity skills, have been linked to physical activity participation (44, 45), designing programs that promote youth confidence and competence in the activity is crucial. Our intervention, promoting manageable activity (i.e., a comfortable intensity at graduated duration intervals) monitored by use of the RPE, supports Pender, Bar-or, Wilk, and Mitchell’s findings (46) with females ages 8–17 years. Youth perceptions of competence may be facilitated through the promotion of environments in which self-referenced standards and indicators of improvement are adopted as opposed to competitive situations in which evaluated outcomes are dependent upon the performance of others (21). Promoting exercise that is challenging but manageable, building confidence for sustaining the activity, is a strategy to increase physical activity. Being able to test out new skills with trusted others is an important part of building competence, especially as youth are experiencing changes due to maturation. Our program results suggest that youth were responsive to the self-rated perception of effort and chose to maintain an activity level that was consistent with a training effect even when the recommended minutes of activity were not recorded.

In a recent review of research promoting girls’ physical activity (47) 14 of 21 interventions were based on a behavioral theory, with only one study (48) describing an intervention held in the classroom for middle school girls. Moreover, the most common outcome for measuring impact of interventions has been weight, BMI, or performance based on the 20-m pacer fitness test. While these measures do have merit the use of the RPE scale to determine improvements in cardiovascular fitness may be more readily accepted by youth since no one is exposed to being the first to drop-out of a test or protocol. Even interventions which used behavior theories did not typically include both activity and didactic sessions, which may aid in the sustainability of the behavior. The current study provides a model for establishing program variables to positively influence increases in relatedness, competence, and autonomy for youth leading to greater physical activity participation.

We found that youth engagement was linked to enjoyment, the non-competitive nature of the activities, having choices of activities, confidence in the activity, and being with friends. In an analysis of community programs for youth, social interaction and enjoyment were common reasons for participation in physical activity while challenges to identity such as having to be active around others who may be judgmental of an unfit body, lacking confidence, and competence were seen as barriers (49). While the current program included both males and females the non-competitive nature of the program and support for all ability levels provided an environment for engagement unlike some physical education classes where females may be marginalized or disruptive behavior may detract from participation (50). The choice of activities promoted youth autonomy and led to greater likelihood of engaging in sustainable activity patterns, especially when those activities were viewed as relevant by peers and supportive others.

The design and results of this intervention add to an emerging body of literature on the use of the principles of relatedness, competence, and autonomy when designing youth physical activity programs in the community. Designing programs with these elements may lead to greater participation and long-term engagement. By using existing social structures for health message delivery, this intervention also works to reinforce healthy lifestyle behaviors for youth, especially in the urban environment.

In order to develop similar interventions with a high likelihood of youth involvement, it will be important to understand the degree to which each of the factors that we have identified influences youth participation and whether one is more critical than the others. To that end, we recommend research that focuses on the degree to which program variables account for youth involvement or how intervention components might be designed to mitigate problems associated with less effective youth programs.

### Limitations

Several factors limit the generalizability of this study. First, the small numbers of youth in this pilot study, which also lacks a control group, must be noted. Results reported here may be due to chance enrollment of certain types of participants, which may have been influenced in unpredictable ways. Second, the study relied in part on self-reported data. However, the trustworthiness of findings was strengthened by using multiple data collection methods. Finally, we have limited data regarding the sustainability of the intervention.

## Conflict of Interest Statement

The author declares that the research was conducted in the absence of any commercial or financial relationships that could be construed as a potential conflict of interest.
